# Highly Effective Conductance Modulation in Planar Silicene Field Effect Devices Due to Buckling

**DOI:** 10.1038/srep14815

**Published:** 2015-10-06

**Authors:** Feras Al-Dirini, Faruque M. Hossain, Mahmood A. Mohammed, Ampalavanapillai Nirmalathas, Efstratios Skafidas

**Affiliations:** 1 Department of Electrical and Electronic Engineering, University of Melbourne, VIC 3010, Australia; 2 Centre for Neural Engineering (CfNE), University of Melbourne, VIC 3010, Australia; 3 Victorian Research Laboratory, National ICT Australia (NICTA), West Melbourne, VIC 3003, Australia; 4 Electrical Engineering Department, Princess Sumaya University for Technology, Amman, Jordan; 5 Institute for a Broadband Enabled Society, University of Melbourne, VIC, 3010, Australia

## Abstract

Silicene is an exciting two-dimensional material that shares many of graphene’s electronic properties, but differs in its structural buckling. This buckling allows opening a bandgap in silicene through the application of a perpendicular electric field. Here we show that this buckling also enables highly effective modulation of silicene’s conductance by means of an in-plane electric field applied through silicene side gates, which can be realized concurrently within the same silicene monolayer. We illustrate this by using silicene to implement Self-Switching Diodes (SSDs), which are two-dimensional field effect nanorectifiers realized within a single silicene monolayer. Our quantum simulation results show that the atomically-thin silicene SSDs, with sub-10 nm dimensions, achieve a current rectification ratio that exceeds 200, without the need for doping, representing a 30 fold enhancement over graphene SSDs. We attribute this enhancement to a bandgap opening due to the in-plane electric field, as a consequence of silicene’s buckling. Our results suggest that silicene is a promising material for the realization of planar field effect devices.

Graphene has long been envisaged as a promising candidate for post-CMOS electronics. However, graphene lacks an electronic bandgap, making it difficult to turn a graphene device off effectively by means of an electric field. Accordingly, many efforts have been redirected towards finding other two-dimensional materials that have an electronic bandgap but still exhibit many of graphene’s desirable electronic properties, such as its high electron mobility. Amongst numerous candidates, silicene, a new material composed of a monolayer of silicon atoms, has attracted the interest of theorists^1^,^2^,^3^,^4^ and more recently experimentalists^5^,^6^,^7^,^8^,^9^,^10^ as a promising material for next generation electronics^11^ and spintronics^12^,^13^. It shares many of graphene’s electronic properties^14^ and exhibits a similar hexagonal structure. However, silicene differs from graphene, which is completely flat, in the fact that it is buckled^15^,^16^,^17^. Due to this buckling, it has been predicted through Density Functional Theory (DFT) calculations that a band gap can be opened in silicene through the application of a perpendicular electric field^18^,^19^, a capability that is only possible in bi-layer graphene^20^. This however, requires external gating and the deposition of other materials on silicene, adding more complexity to the already challenging synthesis process of silicene and degrading some of its desirable electronic properties. In order to overcome such challenges, it is highly desirable to be able to introduce a bandgap in silicene and effectively control its conductance by means of an in-plane electric field, which can be applied through planar two-dimensional device geometries. A unique class of planar field effect devices is the Self-Switching Diode (SSD)^21^, which is a two-dimensional nanoscale rectifier that achieves rectification by means of a self-induced in-plane field effect that acts within the same plane of conduction, making the device completely two-dimensional. This two-dimensional architecture of SSDs, shown in Fig. 1(a–c), makes them very well suited for two-dimensional materials. We had recently proposed their realization on Graphene^22^,^23^, reporting promising performance^22^ and unique negative differential resistance capabilities^23^, and more recently, experimental work has demonstrated graphene SSDs as promising zero bias microwave detectors^24^.

Here we implement SSDs using silicene and study their transport properties using Non-Equilibrium Green’s Function (NEGF) formalism^25^ and the Extended Huckel (EH) method^26^, comparing their performance with graphene SSDs, in order to investigate the effect of an in-plane electric field on silicene and how it compares with graphene.

Our results suggest a similarity between the electronic properties and behaviour of silicene SSDs and graphene SSDs, but show superior performance of silicene SSDs, up to a 30 fold enhancement in rectification ratio, suggesting that an in-plane electric field has much stronger control on the conductivity of silicene nanoribbons in comparison to graphene nanoribbons. We attribute this enhanced performance in silicene SSDs to a bandgap opening that results due to silicene’s buckled structure, as will be discussed later on in the discussion section. Our findings suggest that silicene could potentially be a very promising material for the realization of planar field effect devices.

In the next section we present the simulation results for the transport properties of silicene SSDs followed by their discussion in the final discussion section. The calculation method is described separately in the methods sections at the end of the paper.

## Results

**Figure 1:**
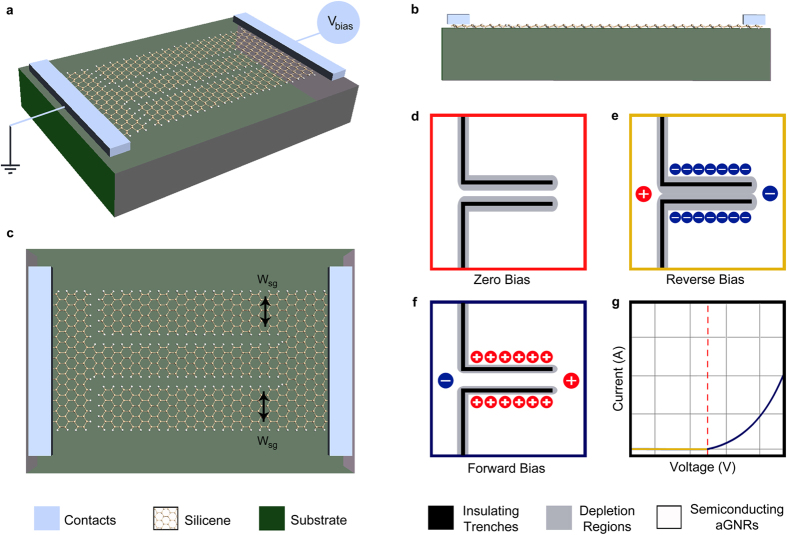
Geometry and principle of operation of Silicene SSDs. (**a**) Perspective view of a Silicene SSD, showing the geometry of the device and the voltage bias direction. (**b**) Side view showing the buckling property of silicene. (**c**) Top view showing the two L-shaped trenches that define the nano-channel of the device and its surrounding two side gates, which have a width of W_sg_ atoms (8 atoms in this case). Schematic diagrams illustrating the principle of operation of a SSD at (**d**) zero bias, (**e**) reverse bias and (**f**) forward bias. (**g**) The expected I-V characteristics of a standard SSD.

A self-switching diode is formed by etching two L-shaped trenches through a two-dimensional material. These two L-shaped trenches define a nanoribbon in between them, which acts as the nano-channel of the device, through which conduction occurs. This nano-channel is surrounded by two side gating nanoribbons, which are used to apply a self-induced electric field onto the channel in order to control its conductance. The two side gates are illustrated through Fig. 1(c), while the principle of operation of a SSD is illustrated through the schematics in Fig. 1(d–g).

With no bias voltage applied to the device, as shown in Fig. 1(d), natural depletion regions form at the edges of the insulating trenches, due to the repulsion between electrons on either side of the trench. When a negative bias voltage is applied to the device, as shown in Fig. 1(e), negative charges accumulate within the side gates, repelling more electrons from the channel and widening the depletion regions within it, eventually pinching it off and preventing conduction through it. On the other hand, when a positive bias is applied to the device, as shown in Fig. 1(f), the depletion regions are narrowed down due to the accumulation of positive charges within the side gates, which attract electrons, widening the channel and increasing current flow through it. The expected I-V characteristics for a SSD are shown in Fig. 1(g).

Since the channel in a silicene SSD is a nanoribbon, it can have either armchair or zigzag edges depending on the orientation of the etched L-shaped trenches. The channel of the device illustrated in Fig. 1(a,c) is an armchair nanoribbon. Silicene SSDs with a zigzag nanoribbon channel can be realized by etching the L-shaped trenches in an orientation that is perpendicular to their orientation in Fig. 1(a,c). However, zigzag nanoribbons are expected to have metallic behaviour and hence are not suited for the channel of a SSD, as it needs to be semiconducting in order to be able to modulate its conductance. A brief study on the performance of silicene SSDs with zigzag nanoribbon channels is presented in the supplementary information provided with this paper. The study indeed confirms that silicene SSDs with zigzag nanoribbon channels do not achieve rectification. This is consistent with DFT calculations and previous predictions^1^, and consistent with graphene SSDs with zigzag nanoribbon channels^22^. Accordingly, in the rest of the paper, all investigated silicene SSDs are chosen to have armchair nanoribbon channels. The side-gating nanoribbons would unavoidably have similar edges to those of the channel, and hence they will have to also be armchair as in Fig. 1(a,c).

### Silicene Self-Switching Diodes

**Figure 2:**
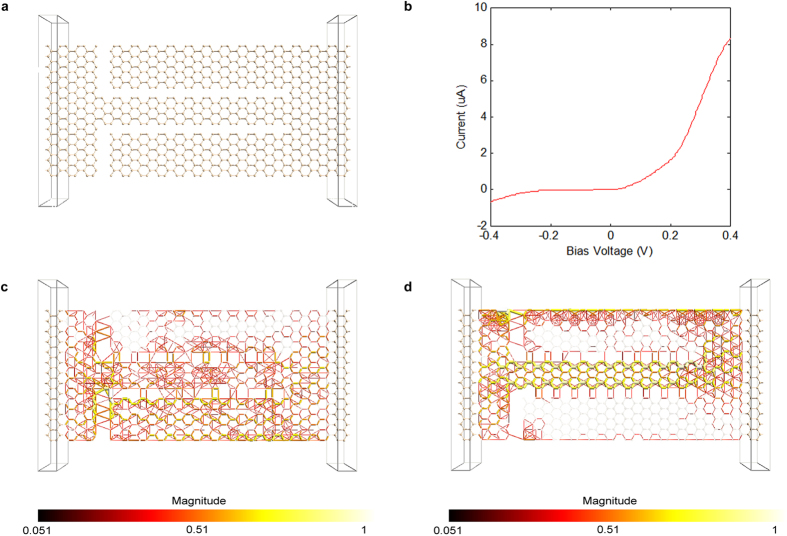
A Silicene SSD with a semiconducting armchair nanoribbon channel that is 6 atoms wide and armchair nanoribbon side gates that are 9 atoms wide. (**a**) The structure of the device. (**b**) I-V characteristics of the device. Transmission pathways plots of the device under (**c**) a reverse bias voltage of −1 V and (**d**) a forward bias voltage of 1 V illustrate how the channel’s conductivity is controlled by the in-plane electric field, turning it off under reverse bias in (**c**) and making it conduct heavily under forward bias in (**d**). The color of a line resembles the magnitude of the local transmission component along a bond according to the color bar at the bottom of each figure. In (**c**,**d**) the atoms and the bonds in the central region of the device geometry are drawn with 90% transparency to allow better visualization of the transmission pathways.

In this sub-section we investigate the transport properties of silicene SSDs with armchair nanoribbon channels. Figure 2(a) shows a silicene SSD with an armchair nanoribbon channel that is 6 atoms wide and side gating nanoribbons that are 9 atoms wide. The I-V characteristics of the device, shown in Fig. 2(b), exhibit significant asymmetry, and achieve noticeable rectification. Transmission pathways plots under reverse and forward biases are shown in Fig. 2(c,d) respectively. Under a reverse bias voltage of −1 V, Fig. 2(c), the device channel does not have an available transmission pathway, confirming its closure. Under a forward bias voltage of 1 V, Fig. 2(d), the channel exhibits strong continuous transmission pathways, confirming its opening. The observed differences between the transmission pathways under reverse and forward biases confirm the strong control that the in-plane electric field has on the semiconducting armchair silicene nanoribbon channel.

**Figure 3:**
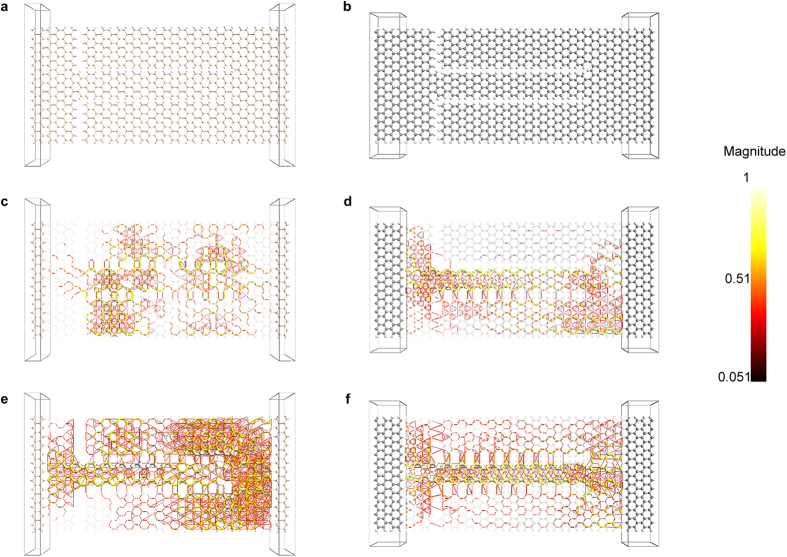
Comparison between silicene and graphene SSDs with semiconducting H-passivated armchair nanoribbon channels. (**a**) Silicene and (**b**) graphene SSDs with armchair nanoribbon channels that are 6 atoms wide and armchair nanoribbon side gates that are 9 atoms wide. The central regions in the two devices are similar; however, the semi-infinite electrodes in (**b**) are longer than in (**a**) due to graphene’s smaller lattice constant, which requires at least two units cells in order to ensure that the electrodes are longer than 6 Å. Transmission pathways plots for (**c**) the silicene and (**d**) the graphene SSDs under a reverse bias voltage of −1 V, showing how the channel is turned off much more efficiently in a silicene SSD. Transmission pathways plots for (**e**) the silicene and (**f**) the graphene SSDs under a forward bias voltage of 1 V, showing how the channel is turned on in both devices. The color of a line resembles the magnitude of the local transmission component along a bond according to the color bar at the side of the figure. In (**c**–**f**) the atoms and the bonds in the central region of the device geometries are drawn with 90% transparency to allow better visualization of the transmission pathways.

One important observation in Fig. 2(c) is the significant transmission pathways that are observed across the vertical insulating trenches, which suggest significant unwanted tunnelling current under reverse bias. This large tunnelling current is predominantly due to the presence of dangling bonds at the edges of the nanoribbons. In order to minimize it, all dangling bonds at the edges of the nanoribbons were passivated with hydrogen, as shown in Fig. 3(a). The transmission pathways plots for the hydrogen passivated device under reverse and forward biases are shown in Fig. 3(c,e) respectively. The plot of Fig. 3(c) confirms the minimization of tunnelling current through the vertical insulating trenches, and illustrates how the channel is strongly turned off in a silicene SSD under reverse bias, while the plot of Fig. 3(e) confirms that the channel is conducting strongly under forward bias.

The presented findings on silicene SSDs with armchair channels are consistent with our previous findings for graphene SSDs with armchair channels^22^. However, in order to investigate how silicene and graphene compare with each other, a graphene SSD with a geometry similar to the silicene SSD of Fig. 3(a) was constructed, and is shown in Fig. 3(b). The transmission pathways plots for the device under reverse and forward biases are shown in Fig. 3(d,f) respectively. Under forward bias, the channels of both devices open up and conduct strongly for silicene and graphene (Fig. 3(e,f)), respectively. However, under reverse bias, the transmission pathways within the graphene device (Fig. 3(d)) show only a slight suppression towards the end of the channel, unlike the complete channel turn-off observed for the silicene device (Fig. 3(c)). This confirms that the silicene channel is more efficiently turned-off than the graphene channel.

In this next subsection we investigate a special class of silicene SSDs; the all-silicene self-switching MISFED, Metal-Insulator-Semiconductor Field Effect Diode.

### All-Silicene Self-Switching MISFEDs

In graphene, armchair nanoribbons have a unique property, in which the bandgap of the nanoribbon varies with its width^27^,^28^. Armchair graphene nanoribbons with a width of 3*p* and 3*p* + 1 atoms, where *p* is an integer, have sizable bandgaps making them semiconducting^28^, while those with a width of 3*p* + 2 atoms have vanishingly small bandgaps, making them behave as if they were metallic^28^.

By utilizing the property of being able to tune armchair graphene nanoribbons from metallic to semiconducting by width variation, we had previously proposed the all-graphene self-switching MISFED (Metal-Insulator-Semiconductor Field Effect Diode)^22^, which is a class of graphene SSDs with metallic side-gates, and semiconducting channels, and can be realized by designing the width of the side gates (W_sg_), marked on Fig. 1(c), to be 3*p* + 2 atoms. This class of graphene SSDs achieves superior rectification when compared to the other two classes of graphene SSDs with semiconducting side-gates^22^.

**Figure 4:**
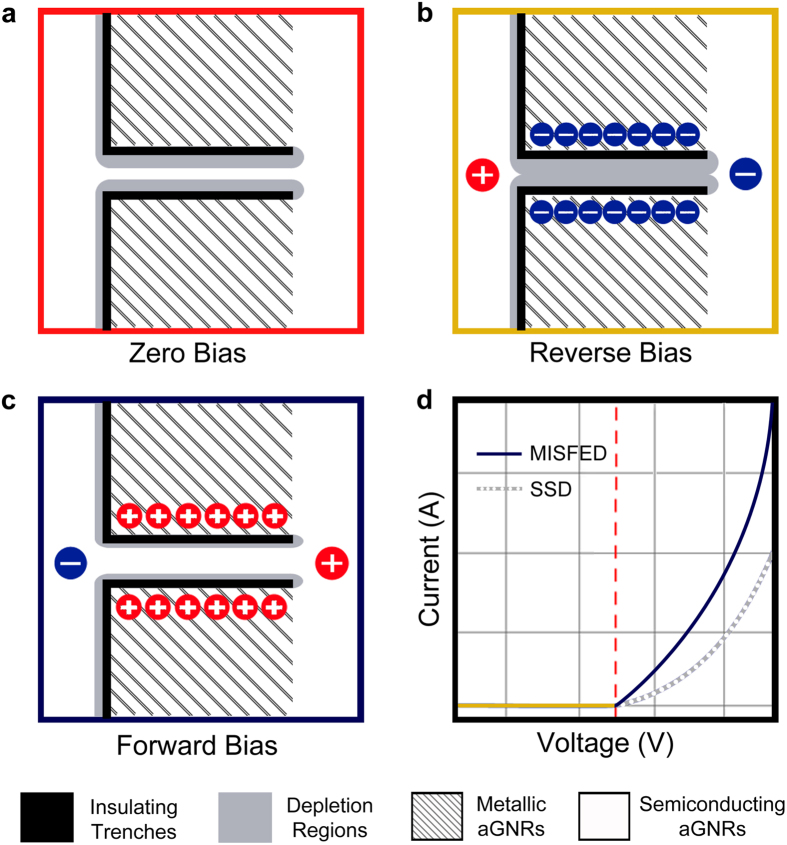
Principle of operation of a MISFED. Schematic diagrams illustrating the principle of operation of a MISFED at (**a**) zero bias, (**b**) reverse bias and (**c**) forward bias. In a MISFED, as shown in the subfigures (**a**–**c**), the depletion regions – grey regions – do not extend into the side gates because they are metallic and only extend into the channel region. (**d**) The expected I-V characteristics of a MISFED (dark blue line) in comparison to those a standard SSD (dotted grey line).

The operation principle of this class of SSDs is illustrated through the schematic diagrams in Fig. 4(a–d). In a MISFED, as shown in the subfigures (a–c), the depletion regions do not extend into the metallic side-gates and only extend into the semiconducting channel region. This makes the effective separation between the side-gates and the channel smaller, enabling stronger control over the channel’s conductivity and resulting in higher forward current and suppressed reverse current, as illustrated through Fig. 4(d).

**Figure 5:**
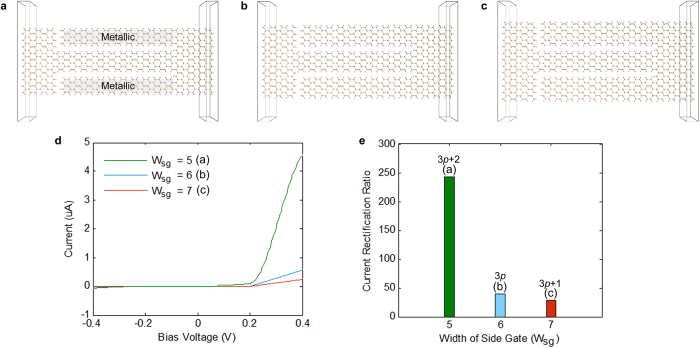
Silicene SSDs with semiconducting H-passivated armchair nanoribbon channels that are 6 atoms wide and armchair nanoribbon side gates that are: (a) 5 atoms, (b) 6 atoms and (c) 7 atoms wide. Hydrogen passivation is at the edges. The side gates of the device in (**a**), highlighted in grey, behave as metallic side gates due to their very small bandgap, making the device behave as a silicene self-switching MISFED. (**d**) I–V Characteristics of the devices in (**a**–**c**) plotted on the same axes in green, blue and red colours respectively. (**e**) A bar chart showing the calculated current rectification ratios of the devices in (**a**–**c**). The current rectification ratio is the maximum ratio of forward current to unwanted reverse current.

Building upon the similarity between graphene and silicene nanoribbons^1^,^29^, it is expected that bandgap tunability with width would also be present in armchair silicene nanoribbons. We investigate this by studying the transport properties of three different silicene SSDs with armchair nanoribbon channels. The three geometries are shown in Fig. 5(a–c), and have similar armchair nanoribbon channels with a width of 6 atoms, while their side gates’ widths (W_sg_), are 5 (3*p* + 2), 6 (3*p*) and 7 (3*p* + 1) atoms respectively. The device in Fig. 5(a) has side gating nanoribbons of the 3*p* + 2 type, which are expected to have metallic behaviour, and therefore, the device is expected to behave in a similar fashion to a graphene self-switching MISFED^22^.

The calculated current-voltage (I–V) characteristics of the three devices are plotted in Fig. 5(d). All three devices show strong rectification confirming the semiconducting behaviour of their armchair silicene nanoribbon channels, and the control that an in-plane electric field has on their armchair nanoribbon channels’ conductivity. Furthermore, the device in Fig. 5(a) shows superior performance, behaving in a similar fashion to the graphene self-switching MISFED. However, a very important unexpected finding seen in Fig. 5(d) is the fact that all types of silicene SSDs, including the ones with semiconducting side-gates (Fig. 5(b,c)), achieve strong rectification. It is postulated that this effect is due to the manner in which silicene nanoribbons interact with an in-plane electric field.

In order to quantify the performance of the three devices of Fig. 5(a–c), the current rectification ratio (the ratio of forward current to unwanted reverse current) was calculated and is shown in Fig. 5(e). Figure 5(e) confirms the previous findings, showing very high current rectification ratios in all three devices, when compared to what can be achieved with graphene without the use of doping. Graphene SSDs with similar geometry to the silicene SSDs in Fig. 5(b,c) do not exhibit significant rectification, with a rectification ratio in the order of 1 (i.e. forward current would equal reverse current)^22^, whilst a graphene SSD (of the MISFED type) similar to the silicene SSD in Fig. 5(a) would achieve a rectification ratio of eight^22^. In order to achieve higher rectification ratios, graphene MISFEDs require nitrogen edge passivation^22^.

In contrast, the achieved rectification ratio for a silicene self-switching MISFED (Fig. 5(a)), without the use of dopants, reaches a value of 240. This is high in comparison to graphene and other types of SSDs realized on 2D materials such as MoS_2_, bulk materials such as compound semiconductor heterostructures^30^,^31^, zinc oxide thin films^32^ or silicon on insulator^33^.

### Experimental Feasibility

While silicene synthesis and its subsequent stability in air has proven to be a challenge, the recently demonstrated synthesis-transfer-fabrication method named ‘silicene encapsulated delamination with native electrodes’ (SEDNE) process^34^ may prove to be a feasible approach for the experimental realization of silicene SSDs. In this process, silicene is grown epitaxially on an Ag(111) thin film deposited on a mica substrate and then capped *in situ* using Al_2_O_3_. The Al_2_O_3_/silicene/Ag film stack is then transferred to a device substrate and patterned into the device structure using electron beam lithography (EBL) and etching, with the native Ag(111) film patterned into electrodes for contacting the device.

While EBL would not be able to define nanoribbons with atomic precision, narrow nanoribbons with sub-20 nm widths and rough edges can be achieved, and these would behave as semiconducting nanoribbons required to construct the channel of the SSD device. To produce metallic side gates, for the silicene MISFEDs, nanoribbons with a width greater than 50 nm can be implemented for the side gates, which would ensure that they are semi-metallic, achieving stronger control over the channel’s conductivity.

Finally it is worth mentioning that with such a process, the fabricated device may not be stable in air for a long period of time, however, the planar 2D architecture of SSD allows further capping, lamination or deposition of other material layers onto silicene, which may be able to help stabilize the silicene lattice. The challenge of silicene’s stability remains to be an open area that needs further investigation, however promising progress is being made towards achieving this endeavour.

## Discussion

**Figure 6:**
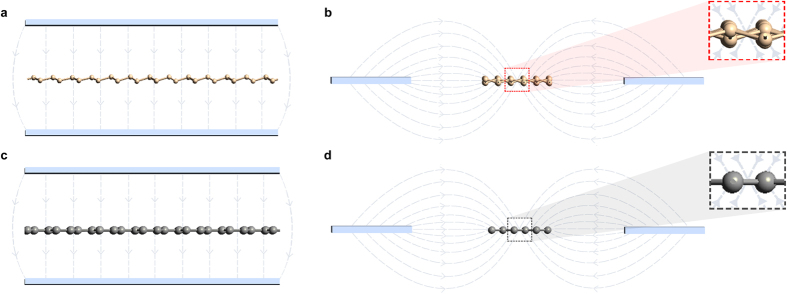
The interaction of perpendicular and in-plane electric fields with silicene and graphene nanoribbons. The two subfigures on the left illustrate the interaction of perpendicular electric fields, applied by top and bottom gates, with: (**a**) a silicene nanoribbon and (**c**) a graphene nanoribbon. The figures show cross-sectional views taken along the length of: (**a**) an armchair silicene nanoribbon and (**c**) an armchair graphene nanoribbon. The two subfigures on the right illustrate the interaction of in-plane electric fields, applied by two in-plane side gates, with (**b**) a silicene nanoribbon and (**d**) a graphene nanoribbon. The figures show cross-sectional views taken along the width of: (**b**) an armchair silicene nanoribbon and (**d**) an armchair graphene nanoribbon. The two subfigures include zoomed-in portions in order to highlight: (**b**) the buckled structure of silicene and how it experiences a net vertical electric field component from the applied in-plane electric field, and (**d**) the completely flat structure of graphene and how it experiences no net vertical electric field component from the applied in-plane electric field.

Silicene shares many of graphene’s electronic properties, but differs in the fact that it exhibits a buckled structure. When a perpendicular electric field is applied to silicene, as shown in Fig. 6(a), an electronic bandgap is observed and can be tuned by varying the applied electric field strength. When a similar perpendicular electric field is applied to graphene, as shown in Fig. 6(c), no bandgap change is observed and only a Fermi level shift occurs.

When an in-plane electric field is applied to an armchair silicene nanoribbon by two side gates with equal voltage, as in a SSD, the electric field lines between the side gates and the channel would have the distribution shown in Fig. 6(b), whilst for an armchair graphene nanoribbon the distribution would be as in Fig. 6(d). In both cases, the electric field lines are not straight and interact with the nanoribbon at an angle, giving them a transverse as well as a vertical component. For the case of flat graphene, the vertical components from the field lines above the nanoribbon are cancelled by the vertical components from the field lines below the nanoribbon, resulting in no net vertical component (Fig. 6(d)). For the case of silicene, these two vertical components of the electric field do not meet at a midpoint, due to silicene’s buckling, and do not cancel out completely. This results in a net vertical electric field component, similar to that of Fig. 6(a), which opens up a bandgap in the nanoribbon and allows it to be turned-off efficiently. Varying the strength of this electric field, allows for bandgap tunability, and leads to effective field effect control over silicene’s conductivity.

In summary, we have investigated the effect of an in-plane electric field on silicene and showed that it results in strong control over its conductivity. The control that is achieved by the in-plane electric field on silicene is much stronger than what can be achieved on graphene due to the buckled structure of silicene, which allows bandgap tunability. We have demonstrated how this effect can be utilized in order to realize high performance planar silicene self-switching diodes. Quantum simulation results show that silicene SSDs can achieve current rectification ratios that exceed 200, without the need for doping or special passivation. This is a 30 fold improvement over what can be achieved with similar graphene devices. Our findings suggest that silicene might offer an attractive platform for the realization of next generation planar field effect devices.

## Methods

In order to capture silicene’s buckling property, its atomic structure was optimized using the Density Functional Theory (DFT) as implemented in Atomistix Toolkit (ATK) package^35^. The Generalized Gradient Approximation (GGA) with the Perdew-Burke-Ernzerhof (PBE) functional was adopted to describe the exchange-correlation interaction^36^. The k-points mesh for the structural optimization was set to 21 × 21 × 1 k-points. The Atomic positions and lattice constant were relaxed until all atomic forces were less than 0.01 eV/Å. A buckling height of 0.5 Å and an optimized lattice constant of 3.86 Å were obtained, showing good agreement with previous studies^1^,^37^,^38^. The optimized structure of silicene is shown in Fig. 1(b). The insulating trenches were then formed in the silicene layer, and, for the hydrogen passivated devices, dangling bonds at the edges were then passivated with hydrogen. This process was conducted for all simulated device geometries prior to transport calculations.

### Transport Calculations Method

Transport calculations were conducted on each device using the Extended Huckel method^26^ and Non-Equilibrium Green’s Function formalism^25^ as implemented in ATK^35^.

The device structure was partitioned as three regions: semi-infinite left electrode (*L*), central scattering region (*C*), and semi-infinite right electrode (*R*). The mesh points in real space calculation were defined as uniformly spaced *k* points of 1 × 10 × 50, with 50 sample points along the transport direction, and 10 points along the width (induced electric field direction). In the used tight-binding model the tight-binding Hamiltonian is parameterized using a two-centre approximation, where the matrix elements are described in terms of overlaps between Slater orbitals at each site. The weighting scheme used for the orbital energies of the offsite Hamiltonian was according to Wolfsburg^39^. Further details about the calculation method can be found in the ATK manual^35^. The electronic transport properties were then calculated using NEGF. Coherent transport of electrons was assumed to occur between (*L*) and (*R*) with Fermi levels μ_L_ and μ_R_ through (*C*) according to the Landauer formula^40^. The coherent current is given by:


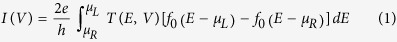


where *T*(*E*, *V*) is the transmission probability of incident electrons with energy *E* from (*L*) to (*R*), *f*_0_(*E* − *μ*_*L*(*R*)_) is the Fermi-Dirac distribution function of electrons in (*L*) and (*R*) respectively, and *V* = (*μ*_*R*_ − *μ*_*L*_)/*e* is the potential difference between (*L*) and (*R*).

Transmission probability, *T*(*E*, *V*), is correlated with 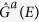 and 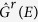, the Green’s function matrices reflected from (*L*) and (*R*) to (*C*) respectively, according to the following equation:


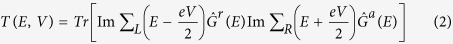


where ∑_*L*(*R*)_ are the electrodes’ self-energies describing coupling with (*C*).

### Calculation of Transmission Pathways

As the Landauer approach only connects the external electrode current *I*(*V*) with the summed energy dependent transmission probability, *T*(*E*, *V*), we need to express local current components at the atomic level along the chemical bonds to describe the variation of coherent electron transport through the system. Local current components may be investigated by extracting local transmission components. The total transmission coefficient can be split into local bond contributions, *T*_*ij*_, which are represented in ATK by lines along the bond lengths, called transmission pathways. The relationship between the total transmission coefficient and the local bond contributions is:


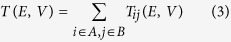


where *A* and *B* represent pairs of atoms separated by an imaginary surface perpendicular along the bond length. The total transmission coefficient is the sum of the local bond contributions between all pairs of atoms *A* and *B*^41^. A negative value of *T*_*ij*_ corresponds to back scattered electrons along the bond, whilst a positive value corresponds to transmitted electrons. The same calculation method was used and is described in greater details in previous work^22^,^23^,^42^.

## Additional Information

**How to cite this article**: Al-Dirini, F. *et al.* Highly Effective Conductance Modulation in Planar Silicene Field Effect Devices Due to Buckling. *Sci. Rep.* **5**, 14815; doi: 10.1038/srep14815 (2015).

## Supplementary Material

Supplementary Information
