# Supplementary material for: Highly Effective Conductance Modulation in Planar Silicene Field Effect Devices Due to Buckling
Source: Sci Rep. 2015 Oct 6;5:14815. doi: 10.1038/srep14815 (PMC4594042; doi:10.1038/srep14815)
Supplement: Supplementary Information [file srep14815-s1.pdf]

Highly Effective Conductance Modulation in Planar Silicene Field Effect Devices Due to Buckling

Supplementary Information

Feras Al-Dirini[1,2,3*], Faruque M. Hossain[1,2], Mahmood A. Mohammed[4], Ampalavanapillai Nirmalathas[1,5] and Efstratios Skafidas[1,2]

[1]Department of Electrical and Electronic Engineering, University of Melbourne, VIC 3010, Australia.

[2]Centre for Neural Engineering (CfNE), University of Melbourne, VIC 3010, Australia.

[3]Victorian Research Laboratory, National ICT Australia (NICTA), West Melbourne, VIC 3003, Australia.

[4]Electrical Engineering Department, Princess Sumaya University for Technology, Amman, Jordan.

[5]Institute for a Broadband Enabled Society, University of Melbourne, VIC, 3010, Australia.

*Address: 203 Bouverie Street, University of Melbourne, VIC 3010, Australia, Email: ferasa@student.unimelb.edu.au,

Zigzag Nanoribbon Channels

Figures S1(a) and (b) show two similar SSDs with zigzag nanoribbon channels 6 atoms wide, and side-gating nanoribbons with a width of 6 and 8 atoms respectively.

The current-voltage (I-V) characteristics of both devices were calculated as described in the methods section, and are shown in Fig. S1(c). Both SSDs do not achieve rectification and are not affected by the in-plane electric field, behaving as metallic nanoribbons. Even when the side gating nanoribbons are wider than the channel of the device, as in Fig. S1(b), the device continues to conduct across the whole bias voltage range and does not achieve any noticeable rectification. This is consistent with DFT calculations and previous predictions[1], and consistent with graphene SSDs with zigzag nanoribbon channels[2].

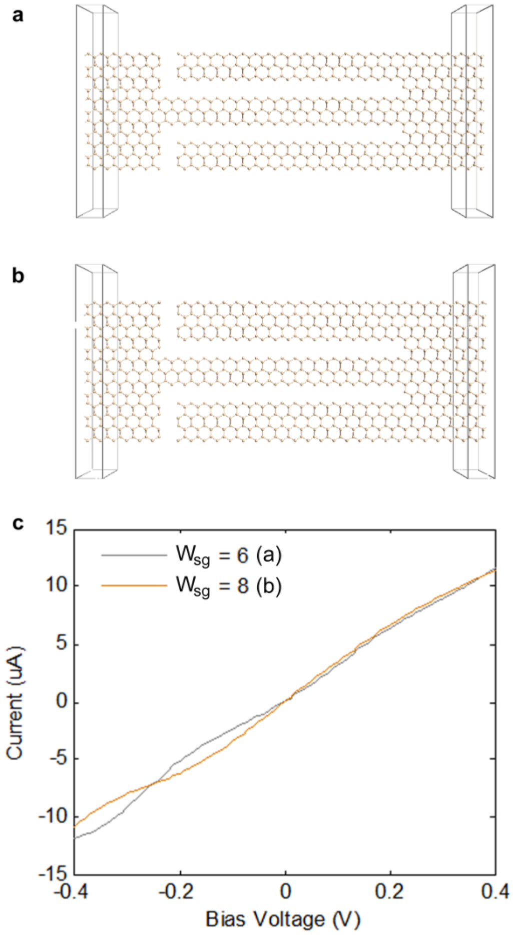

Figure S1 | Silicene SSDs with zigzag nanoribbon channels that are 6 atoms wide and side-gating zigzag nanoribbons that are (a) 6 atoms wide and (b) 8 atoms wide. (c) I-V characteristics of the devices in (a) and (b) plotted on the same axes in grey and brown colours respectively.

To exclude the possibility that conduction is dominated by tunnelling current through the insulating trenches of the devices of Figs. S1(a) and (b), we passivated the dangling bonds at the edges of the nanoribbons with hydrogen, as shown in Figs. S2(a) and (b) respectively. The calculated I-V characteristics of the passivated devices are shown in Fig. S2(c). The devices show no rectification, confirming that the channel is conducting equally under both reverse and forward biases and is not affected by the in-plane electric field.

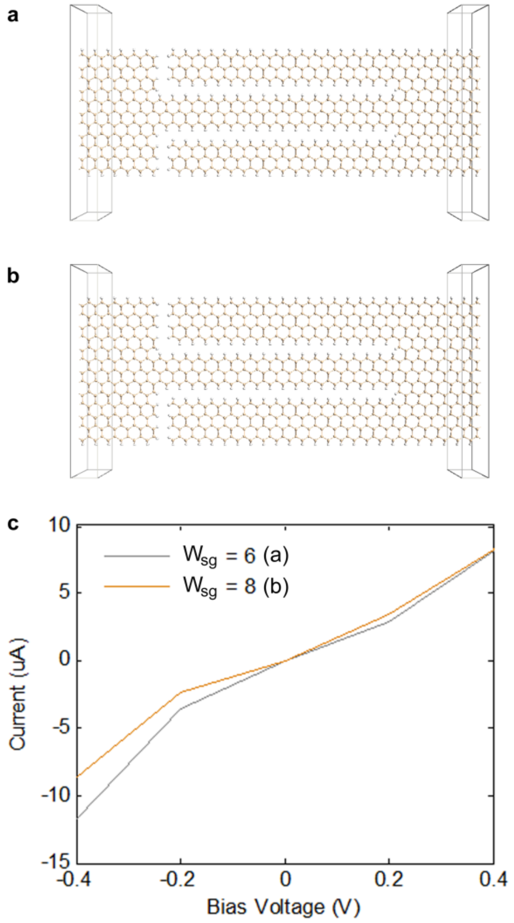

Figure S2 | Silicene SSDs with H-passivated zigzag nanoribbon channels that are 6 atoms wide and side-gating zigzag nanoribbons that are (a) 6 atoms wide and (b) 8 atoms wide. (c) I-V characteristics of the devices in (a) and (b) plotted on the same axes in grey and brown colours respectively. Hydrogen passivation is at the edges of the nanoribbons.

References

1 Cahangirov, S., Topsakal, M., Aktürk, E., Şahin, H. & Ciraci, S. Two- and one-dimensional honeycomb structures of silicon and germanium. *Phys. Rev. Lett.* **102**, 236804 (2009).

2 Al-Dirini, F., Hossain, F. M., Nirmalathas, A. & Skafidas, E. All-graphene planar self-switching MISFEDs, metal-insulator-semiconductor field-effect Diodes. *Sci. Rep.* **4**, 03983 (2014).
